# Quantification of tRNA m^1^A modification by templated-ligation qPCR

**DOI:** 10.1261/rna.079895.123

**Published:** 2024-06

**Authors:** Wen Zhang, Hankui Chen, Marek Sobczyk, Daniel Krochmal, Christopher D. Katanski, Mahdi Assari, Amy Chen, Yichen Hou, Qing Dai, Tao Pan

**Affiliations:** 1 Department of Biochemistry and Molecular Biology; 2 Department of Chemistry, The University of Chicago, Chicago, Illinois 60637, USA

**Keywords:** 1-methyladenosine (m^1^A), SplintR ligase, real-time PCR (qPCR), tRNA, template ligation

## Abstract

N1-methyladenosine (m^1^A) is a widespread modification in all eukaryotic, many archaeal, and some bacterial tRNAs. m^1^A is generally located in the T loop of cytosolic tRNA and between the acceptor and D stems of mitochondrial tRNAs; it is involved in the tertiary interaction that stabilizes tRNA. Human tRNA m^1^A levels are dynamically regulated that fine-tune translation and can also serve as biomarkers for infectious disease. Although many methods have been used to measure m^1^A, a PCR method to assess m^1^A levels quantitatively in specific tRNAs has been lacking. Here we develop a templated-ligation followed by a qPCR method (TL-qPCR) that measures m^1^A levels in target tRNAs. Our method uses the SplintR ligase that efficiently ligates two tRNA complementary DNA oligonucleotides using tRNA as the template, followed by qPCR using the ligation product as the template. m^1^A interferes with the ligation in specific ways, allowing for the quantitative assessment of m^1^A levels using subnanogram amounts of total RNA. We identify the features of specificity and quantitation for m^1^A-modified model RNAs and apply these to total RNA samples from human cells. Our method enables easy access to study the dynamics and function of this pervasive tRNA modification.

## INTRODUCTION

N1-methyladenosine (m^1^A) is among the most abundant eukaryotic RNA modifications present in tRNA, rRNA, and mRNA. In humans, m^1^A is present in all cytosolic tRNA at position 58 (m^1^A58), in 15/22 mitochondrial tRNAs at position 9 (m^1^A9), and in mRNA (Clark et al. 2016; Dominissini et al. 2016; Li et al. 2016; Oerum et al. 2017; Suzuki et al. 2020; Xiong et al. 2023). In tRNA, m^1^A plays a role in its stability and in translational fine-tuning (Liu et al. 2016; Zhang and Jia 2018; Xiong et al. 2023) and has other functions (Oerum et al. 2017). As exemplars, m^1^A58 in cytosolic tRNA^Lys^(UUU), the essential RNA primer for HIV replication, can affect the reverse transcription (RT) fidelity and efficacy in HIV-1 infections (Auxilien et al. 1999). m^1^A58 in yeast tRNA^iMet^ is required for its maturation and stability (Anderson et al. 1998). m^1^A9 of mitochondrial tRNAs is crucial for the correct folding such as in human mitochondrial tRNA^Lys^ (Helm et al. 1998, 1999) and binding to elongation factors (Sakurai et al. 2001, 2005). m^1^A58 can also be reversed by three human eraser enzymes (Liu et al. 2016; Wei et al. 2018; Chen et al. 2019). m^1^A modification levels in specific tRNAs can be used as biomarkers for clinical prognosis, such as the development of COVID-19 severity (Katanski et al. 2022). Thus, the ability to quantify m^1^A modification rapidly and quantitatively in individual tRNA would be a valuable tool for mechanistic studies and diagnostic applications.

m^1^A modifications in RNA are commonly measured using mass spectrometry (MS), thin layer chromatography (TLC), or reverse transcription (RT). LC–MS and TLC methods generally measure the total m^1^A levels in bulk RNA; an individual tRNA m^1^A level can be measured after its isolation from bulk RNA (Suzuki and Suzuki 2014; Suzuki et al. 2020), which can be laborious, time consuming, and requires a large amount of material. RT measures m^1^A in individual tRNA through an m^1^A-induced stop in primer extension using low processive reverse transcriptases, which can be quantified by gel electrophoresis (Saikia et al. 2010; Clark et al. 2016). The RT method can also be used in high-throughput sequencing by quantifying the “mutated” and/or “stopped” reads at the tRNA m^1^A site induced by the readthrough of the m^1^A nucleotide by high processive reverse transcriptases. Mutational profiling with sequencing (MaP-seq) has been widely used to study m^1^A and multiple other modifications in RNA (Hauenschild et al. 2015; Clark et al. 2016; Zubradt et al. 2017). Although powerful, sequencing of tRNA is still slow to implement, expensive, and requires tens of nanograms of the input RNA.

Here, we describe a templated-ligation and quantitative PCR (qPCR) method to quantify m^1^A modification at single base resolution. qPCR is a routine and high-sensitivity method to study specific RNA properties. For quantitation, the RNA needs to be converted into cDNA using reverse transcriptase (RT-qPCR), or the RNA is used as the template to ligate two DNA oligonucleotides followed by PCR of the ligation product (Jin et al. 2016; Krzywkowski and Nilsson 2017; Yeakley et al. 2017). Because of the high extent of its modifications and structure, RT of tRNA is generally not sufficiently robust for RT-qPCR measurements. Furthermore, our goal is to target m^1^A modification at specific sites which may not have cDNA signatures that can be readily distinguished by qPCR. We therefore adapted a templated-ligation (TL-qPCR) approach using the highly efficient SplintR ligase, which was first used for miRNA studies (Jin et al. 2016; Krzywkowski and Nilsson 2017) and m^6^A modification detection in mRNAs (Xiao et al. 2018). We show that SplintR ligation works well for tRNA with subnanogram amounts of total RNA. More importantly, we develop this method for the new application to quantify the levels of m^1^A modification in specific tRNA in total RNA samples.

## RESULTS AND DISCUSSION

### Principles of templated-ligation qPCR (TL-qPCR) assessment of m^1^A modification

tRNA is the most abundant RNA species in a total RNA sample by molarity. tRNA is also extensively modified with over 100 various modifications in three kingdoms of life. Current methods of detecting tRNAs and tRNA modifications include LC/MS (Suzuki and Suzuki 2014; Zhang et al. 2022), northern blot (Zhang et al. 2020; Khalique et al. 2022), next-generation sequencing (Cozen et al. 2015; Zheng et al. 2015; Clark et al. 2016), and end-ligation-based qPCR (Honda et al. 2015b). A SplintR ligation-based qPCR method has been developed to detect and quantify microRNA (miRNA) (Jin et al. 2016; Krzywkowski and Nilsson 2017). This method relies on splint ligation by the SplintR ligase using miRNA as the template. The ligation efficiency of template splint ligation by SplintR is very high, in contrast to previous methods using T4 RNA ligase 2 or T4 DNA ligase. In addition, the ligation efficiency of SplintR is negligible without the template. Thus, the ligation efficiency is proportional to the abundance of the miRNA.

**FIGURE 1.**
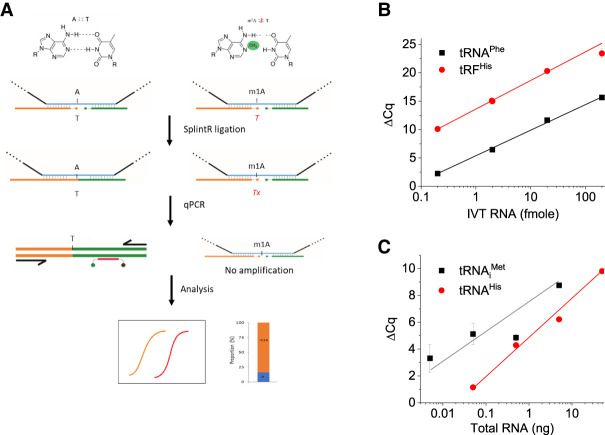
TL-qPCR schematics and quantitative and sensitive detection of synthetic and biological tRNAs. (*A*) The basic design of measuring m^1^A in tRNA. The 5 linkers (5lkrT) with phosphorylated T at the 5′ end binds to complementary RNA template containing A or m^1^A at the same position. SplintR ligase ligates the 5lkrT linker and the 3′ linker at the joint position where the T pairs with A, but not with m^1^A. The ligated 5lkrT product is then used as template in qPCR using fluorescent probes. Comparing the difference of *Cq* value from A and m^1^A after normalization to controls, the m^1^A levels in the RNA sample are obtained. (*B*) Detection of model tRNA. Δ*Cq* value between RNA sample and water control using 0.2–200 fmol in vitro transcript of yeast tRNA^Phe^ (black) or a synthetic human tRNA^His^ fragment (red) using the 5lkrT linker in ligation. Δ*Cq* values have a nice linear correlation with the log values of input amounts. (*C*) Detection of human tRNA_i_^Met^ and tRNA^His^ in HEK293T total RNA. Δ*Cq* value between RNA sample and water control. tRNA_i_^Met^ or tRNA^His^ is still detectable at 5 or 50 pg total RNA input, respectively.

We took advantage of the SplintR ligation selectivity and developed a templated-ligation followed by qPCR assay for the detection and quantitation of m^1^A modification in tRNA (Fig. 1A). A previous method termed “SELECT” for m^6^A modification detection relies on the ability of m^6^A to inhibit both RT and splint ligation (Xiao et al. 2018). We reasoned that the methyl group at the N1 position of m^1^A affects the base-pairing between A and T, which may block the template splint ligation by SplintR and may be sufficient to distinguish A from m^1^A without RT. We designed two pairs of linker oligos that bind to the tRNA template near or far away from the m^1^A site. For the linker oligos near the modification site, the 5′ end of the upstream linker (5′ linker, 5lkr) has a phosphorylated T that binds to the complementary template containing A or m^1^A and a PCR primer-binding site. The downstream linker (3′ linker, 3lkr) has a fluorescent probe binding site and a PCR primer-binding site. To examine the feasibility of our method at the m^1^A site, we also included an optional AlkB demethylase treatment (Cozen et al. 2015; Zheng et al. 2015) step for the same sample that converts m^1^A to A. After SplintR ligation, the amount of ligated product can be detected by qPCR with fluorescent probes. The m^1^A modification fraction can be calculated using Equation 1 or 2 (see below) based on the qPCR threshold (*Cq*) values of the two SplintR ligation samples using linker oligos that bind to the tRNA near or away from the m^1^A site.

To test the sensitivity of the TL-qPCR method for the quantification of small RNAs in the absence of m^1^A modification, we used the in vitro transcript of yeast tRNA^Phe^, a synthetic RNA oligonucleotide corresponding to the human 5′ tRNA^His^ fragment (Martinez et al. 2017; Wang et al. 2018), and tRNA^iMet^ in total human RNA. The Δ*Cq* value between the RNA sample and water control of the two unmodified RNAs was proportional to the input amount of 0.2–200 fmol (Fig. 1B). Using the human tRF^His^ oligos and probes, we obtained a quantitative relationship of tRNA^His^ and tRNA_i_^Met^ in total human RNA (Fig. 1C), and detected tRNA_i_^Met^ in as little as 5 pg HEK293T total RNA input. These results validate the principle of our TL-qPCR design for the quantitative measurements of native tRNA.

### TL-qPCR quantitation of m^1^A in synthetic oligonucleotides: optimization of linker oligo end sequence, position, and SplintR ligation temperature

**FIGURE 2.**
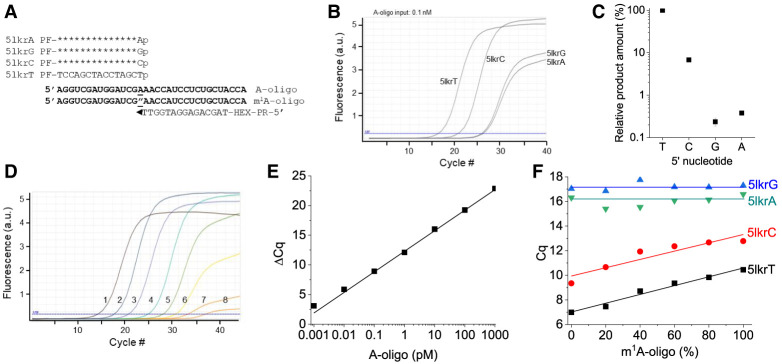
TL-qPCR on human tRNA_i_^Met^ mimicking RNA oligonucleotides. (*A*) The oligos and linkers used in SplintR ligation for tRNA_i_^Met^-m^1^A58. A oligo and m^1^A oligo: two RNA oligos containing A or m^1^A-modified nucleotide (indicated with ″ according to conventional RNA modification nomenclature) as underlined. PF and PR: primer-binding site for PCR. HEX: qPCR fluorescent probe binding site. (*B*) qPCR curves and (*C*) relative PCR product amounts of the four linkers 5lkrT/C/G/A in the templated SplintR ligation using 0.1 nM A oligo as input. (*D*) Amplification curves and (*E*) standard Δ*Cq* curve of the dilution series of the A oligo input (#1 to #7 for 10× serial dilution from 1 nM to 1 fM) with H_2_O as a negative control (#8), generated with the linker 5lkrT in the SplintR ligation. (*F*) Standard *Cq* curves of 1 nM total m^1^A/A oligo mixture inputs in the SplintR ligation using the linker 5lkrT.

To examine the quantitative nature of our TL-qPCR method for m^1^A, we applied it to synthetic RNA oligos that have identical sequences of the m^1^A58 surrounding region of human tRNA_i_^Met^. First, we tested whether 1 nt mismatch at the ligation site affected the TL-qPCR product amount, and by inference, template splint ligation efficiency using unmodified RNA oligo. We designed four different 5′ linker oligos with T, C, G, or A at the 5′ end (Fig. 2A). The TL-qPCR profile showed that a 1 nt mismatch was sufficient to very significantly suppress the template splint ligation by SplintR (Fig. 2B). The relative amounts of PCR product for C, G and A-ending 5′ linker oligos were <10% compared to the T-ending 5′ linker, with C, G, and A-ending mismatched linker oligos at the level of 6.9%, 0.2%, 0.3% of the T-ending linker, respectively (Fig. 2C). This result demonstrates that a 1 nt mismatch can reduce the template splint ligation significantly, as expected. These results are crucial for our TL-qPCR strategy since it is based on the impaired base-pairing between m^1^A and T at the modification site. Next, we tested whether our method could quantify the abundance of the templated ligated product by varying the input amount of the RNA oligo. The qPCR amplification profile of the serial dilution of the unmodified oligo showed that the *Cq* values increased proportionally as the input amount was reduced (Fig. 2D). The standard curve of the serial diluted samples showed an excellent linear correlation between the *Cq* value and the RNA oligo input (*R*^2^= 0.974, Fig. 2E). This result demonstrates that our method can quantitatively measure the template splint ligated product.

**FIGURE 3.**
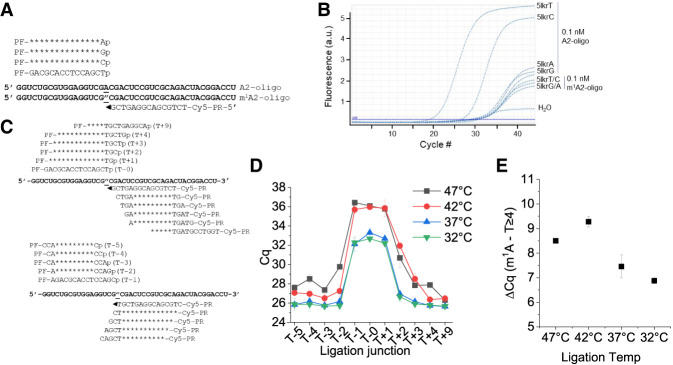
Optimization of TL-qPCR efficiency regarding m^1^A proximity and ligation temperature. (*A*) Oligonucleotides used and ligation oligo design. (*B*) Amplification curves of linker 5lkrT/C/G/A in A and m^1^A oligo in ligation. (*C*) Ligation oligo design at varying ligation sites away from the m^1^A site. (*D*) *Cq* plots as a function of ligation oligos with ligation sites at m^1^A (T + 0) or progressively away from the m^1^A site. (*E*) Δ*Cq* values between the m^1^A site and control site (using average *Cq* values of sites ≥4 nt away from the m^1^A site) under different template splint ligation temperatures.

To investigate the quantitative nature of TL-qPCR for m^1^A levels, we varied the ratio of the synthetic m^1^A-modified oligo and unmodified oligo in the input and performed TL-qPCR with 5′ linker oligos that have T, C, G, or A at the 5′ end. *Cq* values obtained using T-ending 5′ linker oligo showed the best linear correlation (*R*^2^= 0.977, Δ*Cq* of one- to twofold) with the ratio of m^1^A RNA oligo compared to other 5′ linker oligos (Fig. 2F). However, our sample with supposedly 100% m^1^A oligo still showed a low amount of TL-qPCR product, and a low level of ligation product was detectable using this oligo alone (Supplemental Fig. S1A). This result may be derived either from incomplete suppression of ligation by m^1^A modification, the residual presence of unmodified oligo, or a m^1^A-to-m^6^A migration during oligo synthesis (Engel 1975; Liu et al. 2022). ESI-MS showed that the commercial m^1^A oligo did not contain unmodified oligo but was consistent with the presence of m^6^A (Supplemental Fig. S1B). From our TL-qPCR results, we estimate ∼10% m^1^A-to-m^6^A conversion during the commercial m^1^A-oligo synthesis, whereas this conversion was negligible during our TL-qPCR procedure (see Fig. 3 result below). Nevertheless, these results demonstrate that our TL-qPCR method can quantify the m^1^A modification fraction in model oligos.

We further investigated the context dependence of m^1^A effects using another pair of unmodified and m^1^A-modified oligos that were synthesized using a method that minimized m^1^A-to-m^6^A conversion (Zhou et al. 2019). We found very little ligated product using only the m^1^A-modified oligo (Supplemental Fig. S2A), indicating that m^1^A can indeed block templated splint ligation. Furthermore, the ligated product was proportional to the ratio of unmodified oligo in total RNA mixtures. We again performed TL-qPCR with 5′ linker oligos with a different 5′ end using m^1^A-modified or unmodified oligos (Fig. 3A). The *Cq* values with this m^1^A-modified oligo were >10 higher than those with the corresponding unmodified oligo under this condition (Fig. 3B; Supplemental Fig. S2B). Using this fully m^1^A-modified oligo, we also examined how many residues away from the m^1^A site the ligation site can be before m^1^A no longer has an effect on TL-qPCR. This enables us to choose a site for on-target control of TL-qPCR. We shifted the ligation site up to 5 or 9 nt upstream (T − N) or downstream (T + N), respectively, of the target m^1^A site (Fig. 3C). At the same time, we also tested how splint ligation reaction temperature affected the specificity and sensitivity of our method. Temperature optimization can be important for quantifying m^1^A modification in tRNA, a higher temperature could more readily disrupt the tRNA structure, but may decrease SplintR ligase reactivity. When the ligation site was 3–5 nt away either upstream or downstream from the m^1^A site, the *Cq* value differences reached a plateau, and this result was maintained at temperatures between 32°C and 47°C (Fig. 3D). Increasing the ligation reaction temperature above 42°C decreased the TL-qPCR product amounts (Fig. 3D), but also slightly increased the difference between A and m^1^A RNA oligos (Fig. 3E). Based on these results, we generally used 37°C for template splint ligation and the on-target control site ≥4 nt away from the m^1^A site for the biological RNA studies below.

### m^1^A modification in biological tRNAs and rRNA by TL-qPCR

**FIGURE 4.**
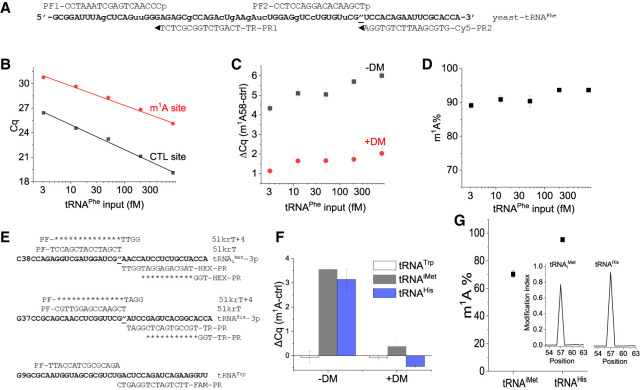
Measuring m^1^A58 modification in biological RNA. (*A*) The sequence of yeast tRNA^Phe^ and oligos used in TL-qPCR for m^1^A58 modification (″: bold and underlined). The two pairs of linkers for yeast tRNA^Phe^-58m^1^A and for -5p (control) contain sequences for Cy5 and TR (Texas Red) probe binding sites for qPCR. (*B*) *Cq* values of m^1^A (red) or control (black) ligation oligos of serial diluted inputs of yeast tRNA^Phe^ from 3.1 to 800 fM without AlkB treatment. (*C*) Differential *Cq* values between m^1^A and control ligation oligos of serial diluted yeast tRNA^Phe^ samples without (black) and with (red) AlkB demethylase (DM) treatment. (*D*) Fraction of m^1^A58 in yeast tRNA^Phe^ as measured by TL-qPCR using varying amounts of input RNA. (*E*) Oligonucleotides and linkers used in SplintR ligation for the m^1^A58 site of human tRNA_i_^Met^ and tRNA^His^, tRNA^Trp^ is used as an internal control. Three-color qPCR is run using either HEX(m^1^A-iMet) + TR(m^1^A-His) + FAM(Trp) or HEX(CTRL-iMet) + TR(CTRL-His) + FAM(Trp) combination. (*F*) Δ*Cq* values of 5lkrT (m^1^A) and 5lkrT + 4 (CTRL) on tRNA_i_^Met^ (HEX probe), tRNA^His^ (TR probe), and tRNA^Trp^ (FAM probe) without (DM−) and with AlkB treatment (DM+). (*G*) m^1^A58 fraction measured by TL-qPCR for tRNA_i_^Met^ and for tRNA^His^. Average of *n* = 3 biological replicates. Inset: modification index (MI) around the m^1^A58 (±5 nt) site measured by DM-tRNA-seq for tRNA_i_^Met^ and tRNA^His^ (sequencing data from NCBI GEO GSE66550).

Next, we applied our method to detect and quantify m^1^A58 modification in yeast tRNA^Phe^. We designed one pair of linker oligos at the m^1^A site and another pair at an on-target site away from the m^1^A site, which served as a m^1^A independent control for that tRNA; these oligos have different fluorescent probe binding sites for multiplex qPCR (Fig. 4A). We performed TL-qPCR on serial diluted yeast tRNA^Phe^ samples. The *Cq* values of both the control and m^1^A ligation sites increased as the input RNA amount decreased (Fig. 4B). To validate that the m^1^A58 targeting oligos were indeed responsive to m^1^A, we partially removed the m^1^A modification using the *Escherichia coli* AlkB demethylase (Zheng et al. 2015) and used the demethylase-treated sample as a template for TL-qPCR. Changes in differential *Cq* values (Δ*Cq*) of the control versus m^1^A site were much smaller in the demethylase-treated sample, confirming that our m^1^A58 targeting oligos indeed reports this modification in yeast tRNA^Phe^ (Fig. 4C). Furthermore, the difference of Δ*Cq* (the control vs. m^1^A site) between the untreated and the demethylase treated (i.e., less m^1^A) (ΔΔ*Cq*) remained approximately the same when varying the input RNA from 800 to 3.1 fM, indicating the robustness of our method for a natural tRNA (Fig. 4C). Assuming the removal of m^1^A by demethylase is complete, we can use the above defined ΔΔ*Cq* values and Equation 1 to calculate the m^1^A modification fraction as follows:

(1) $$\Delta Cq=Cq_{m^{1}A}-Cq_{\mathrm{ctrl}}$$

$$\Delta \Delta Cq=\Delta Cq_{-\mathrm{DM}}-\Delta Cq_{+\mathrm{DM}}$$

$$A\text{\%}=100\text{\%}\times 2^{-\Delta \Delta Cq}$$

$${m\;}^{1}A\text{\%}=100\text{\%}\;-A\text{\%}$$

We obtained the yeast tRNA^Phe^ m^1^A58 modification fraction at a 90%–93% level (Fig. 4D), which is close to the ∼97% measured by primer extension using AMV RT stop and gel electrophoresis (Saikia et al. 2010).

We next tested our method for the m^1^A58 modifications in human tRNAs using HEK293T total RNA as input. Unlike the purified yeast tRNA^Phe^ sample, this experiment tested our ability to directly measure m^1^A in a complex biological mixture. We chose three target tRNAs for our linker oligo design with different fluorescent probe binding regions for the m^1^A sites and an on-target control site (Fig. 4E), which enabled three-color qPCR to measure them simultaneously. For m^1^A sites, we chose to study human tRNA_i_^Met^ and tRNA^His^, which were of biological interest in previous studies (Honda et al. 2015a; Wang et al. 2018); also, tRNA_i_^Met^ m^1^A is a biomarker for COVID symptom severity (Katanski et al. 2022). We also designed the ligation oligos for tRNA^Trp^ at a region with no known m^1^A modification, thus using tRNA^Trp^ in the same sample as an internal sample quality and quantity control. We performed TL-qPCR using 26 ng total RNA with and without demethylase treatment as template. For the control tRNA^Trp^ site, demethylase treatment did not change the Δ*Cq* value, as expected. However, the *Cq* values of the tRNA_i_^Met^ and tRNA^His^ m^1^A sites were significantly reduced after demethylase treatment, demonstrating the presence of m^1^A at these locations (Fig. 4F). Thus, with the help of the internal control (tRNA^Trp^), we can use the simplified equation (Equation 2) below to calculate the fraction of m^1^A modification.

(2) $$\Delta Cq=Cq_{m^{1}A}-Cq_{\mathrm{ctrl}}$$

$$A\text{\%}=100\text{\%}\times 2^{-\Delta Cq}$$

$${m\;}^{1}A\text{\%}\;=100\text{\%}-A\text{\%}$$

With this method, we determined the m^1^A58 fraction for tRNA^iMet^ and tRNA^His^ in the samples without demethylase treatment to be 70 ± 3% and 95 ± 1%, respectively (Fig. 4G). We have shown previously that the DM-tRNA-seq method could quantitatively report m^1^A58 fractions in HEK293T cells, which was 76% and 95% for tRNA^iMet^ and tRNA^His^, respectively (Fig. 4G, inset; Clark et al. 2016). The excellent correlation between TL-qPCR and high-throughput tRNA sequencing demonstrates the effectiveness and accuracy of our TL-qPCR method in detecting and quantifying the m^1^A fraction in tRNA.

We also observed near zero Δ*Cq* values for the m^1^A site and the control site for all three tRNAs studied here when m^1^A was removed by the demethylase treatment (Fig. 4B,C,F), suggesting a similar hybridization efficiency for these oligos to tRNA despite their hybridization to distinct locations in the tRNA structure.

To further test the robustness of TL-qPCR, we applied it to the quantification of m^1^A9 modification in human mitochondrial tRNA^Val^ and m^1^A1322 in human 28S rRNA, using 24 ng of HEK293T total RNA with or without demethylase treatment as input. Mitochondrial tRNA^Val^ also has an m^2^G10 modification next to m^1^A9 (Cappannini et al. 2024), and 28S rRNA has an Am modification 4 nt downstream from m^1^A1322 (Taoka et al. 2018). We designed a pair of linker oligos targeting the m^1^A site, along with another set targeting an on-target site for respective RNA away from the m^1^A site, acting as an independent control for each m^1^A site (Supplemental Fig. S3A). We determined the m^1^A fractions for human mitochondrial tRNA^Val^ m^1^A9 to be 77 ± 2% and for human 28S rRNA m^1^A1322 to be 92 ± 1% (Supplemental Fig. S3B). Previous sequencing results showed 94% m^1^A9 for mt-tRNA^Val^ and over 98% m^1^A1322 for 28S rRNA (Zheng et al. 2015; Clark et al. 2016; Taoka et al. 2018). The consecutive m^1^A9m^2^G10 in mt-tRNA^Val^ may result in a 1.2× under-estimated m^1^A level by TL-qPCR or an over-estimated m^1^A9 level by sequencing.

### Concluding remarks

In summary, we developed a templated splint ligation-based qPCR method for the detection and quantification of m^1^A modifications in tRNA and rRNA. With on-target control and internal control, we can obtain the fraction of m^1^A modifications at a given site. In addition, by including an optional demethylase treatment step, we can also probe the effect of tRNA structure and differential hybridization efficiencies on the quantification of m^1^A modification in highly structured RNA regions. Our method relies on the specificity and sensitivity of the SplintR ligase. A single base mismatch or inefficient binding of the ligation oligos caused by Watson–Crick face modifications is sufficient to vastly reduce or block the template splint ligation by SplintR ligase. Thus, in principle, our method can be adapted to the studies of other Watson–Crick face modifications in RNA such as N1-methylguanosine (m^1^G), N3-methylcytosine (m^3^C), or N2,2-dimethylguanosine (m^2^_2_G) that are also abundant in eukaryotic tRNAs. Other RNA modifications such as pseudouridine (Ψ) may also be studied using TL-qPCR after chemical treatments that generate Ψ adducts interfering with Watson–Crick base-pairing. Our method can quantitatively access m^1^A modification in as low as 3 fmol tRNA or ∼75 pg total RNA. In addition to RNA modification detection, our method can be used to detect the abundance of tRNAs or other small RNA biomarkers using a 10–100 pg range of input total RNA. Our method can be performed in a matter of hours (∼3–4 h), is easy to operate, and requires less hands-on time compared to sequencing or primer extension followed by gel electrophoresis. The ease of using the half-day protocol for target RNA modification or abundance detection should enable widespread application of the TL-qPCR method for tRNA and tRNA modification studies.

## MATERIALS AND METHODS

### Oligonucleotide design

The RNA (A/m^1^A) and DNA oligonucleotides used in the study were purchased from IDT (Integrated DNA Technologies) using standard desalting purification. RNA oligo synthesis that retains m^1^A modification for m^1^A or A containing oligos (A2/m^1^A2) was described in Zhou et al. (2019). Briefly, RNA oligonucleotides were synthesized in-house using an Expedite DNA synthesizer, followed by normal deprotection for regular oligonucleotides and vendor-suggested deprotection for RNA oligonucleotides containing m^1^A modifications to avoid Dimroth rearrangement. After deprotection, the RNA oligonucleotides were purified through HPLC with a C18 column and were eluted with 0%–20% acetonitrile in 0.1 M triethylammonium acetate. The desired peak was collected and dried by lyophilization.

Two sets of SplintR ligation linkers (5′ linkers and 3′ linkers) were designed for each m^1^A site with one set of linker oligos targeting the m^1^A site and the other set targeting an upstream or downstream control region. The 5′ linker oligos were phosphorylated at the 5′ end to enable ligation. A primer-binding sequence for forward or reverse primers during PCR, and a synthetic Taqman probe binding sequence were designed just downstream from the reverse primer-binding site in the 3′ linker oligo.

Synthetic primers and probes were used in qPCR for m^1^A quantification, which were all designed as artificial sequences without complementary matching to any human genomic DNA or RNA sequences. A set of primers and a fluorescence-labeled probe were designed for each m^1^A site to enable multiplex qPCR (Cy5, Texas Red, and FAM).

All DNA and RNA oligo and probe sequences are provided in Supplemental Tables S1–S5.

### HEK293 cell total RNA and demethylase treatment

HEK293T cells were cultured in DMEM medium (Cytiva, SH30022.01) with 10% FBS and 1% Pen–Strep (Penicillin–Streptomycin) to 80% confluency (Zhang et al. 2019). Total RNA was extracted using TRIzol reagent according to the manufacturer's manual. Demethylase treatment was performed as reported previously (Zheng et al. 2015).

### TL-qPCR procedure

TL-qPCR consists of two major steps, template ligation and qPCR. To quantify m^1^A modification, a pair of template ligation reactions targeting the m^1^A site and control site were performed. Briefly, ligation linker oligos for the m^1^A site or control site were mixed at a final concentration of 100 nM as 10× stock solution separately. The amount of the ligation oligos was in large molar excess to the template RNA, so that every template RNA should have both oligos hybridized to it at most of all times. For each template ligation reaction, 1 µL total RNA (typically 10–50 ng) and 1 µL 10× ligation linker oligos solution were added to the PCR tube followed by the addition of 6.8 µL H_2_O. The ligation mixture was briefly vortexed and spun down. The ligation mixture was then incubated in a thermocycler at 90°C for 2 min followed by gradual decreasing to 40°C. One microliter 10×SplintR ligase buffer and 0.2 µL SplintR ligase (NEB, M0375L) were then added to each ligation mixture and mixed (10 µL final ligation mixture). The ligation mixture was incubated at 37°C for 30 min followed by incubation at 68°C for 5 min to deactivate the SplintR ligase. qPCR reactions were performed in 20 µL on a Cielo 6 qPCR machine (Azure Biosystems) with specific primers and probes (Supplemental Tables S1–S5). Briefly, PCR primers and fluorescent probes were premixed as a 10× solution containing 2 µM PCR primers and 1 µM fluorescent probes. For each qPCR reaction, 10 µL 2× PrimeTime Gene Expression Master Mix (IDT, 1055772), 2 µL 10× primer/probe mix, 7 µL H_2_O, and 1 µL ligation mixture were added to the PCR tube and mixed. qPCR reactions were performed on a 96-well qPCR machine (Azure Biosystems, Cielo 6) using the conditions below: initial denaturation, 95°C, 2 min; subsequent denaturation, 95°C, 20 sec; annealing and extension, 56°C, 20 sec, 40 cycles.

*Cq* values were obtained using the qPCR machine software (Azure Biosystems). *Cq* values were then analyzed using the Δ*Cq* and ΔΔ*Cq* method after normalizing to endogenous control, and m^1^A modification fractions were then calculated using either Equation 1 or Equation 2. When data from both with or without demethylase treatment were obtained, we used Equation 1 to calculate the m^1^A modification fraction as follows: (i) Calculate the Δ*Cq* values between the m^1^A site and control site for both untreated (−DM) and demethylase treated (+DM) samples using $\Delta Cq=Cq_{m^{1}A}-Cq_{\mathrm{ctrl}}.\;\Delta Cq$ value for +DM sample served as control to exclude the differential hybridization efficiencies between the m^1^A site and control site, assuming the removal of m^1^A by demethylase was complete. (ii) Calculate the ΔΔ*Cq* values between −DM and +DM samples using ΔΔ*Cq* = Δ*Cq*_−DM_ − Δ*Cq*_+DM_. (iii) Calculate the fraction of A using A% = 100% × 2^−ΔΔ*Cq*^. (iv) Finally, the m^1^A fraction can be obtained using m^1^A% = 100% − A%.

Without the demethylase-treated sample, the simplified Equation 2 and Δ*Cq* value can be used to calculate the m^1^A modification fraction as follows: (i) Calculate the Δ*Cq* value between the m^1^A site and control site using $\Delta Cq=Cq_{m^{1}A}-Cq_{\mathrm{ctrl}}$. (ii) Calculate the fraction of A first using A% = 100% × 2^−Δ*Cq*^. (iii) The m^1^A fraction can be obtained using m^1^A% = 100% − A%.

The entire TL-qPCR procedure without demethylase treatment takes about 3–4 h depending on the number of samples processed.

### TL-qPCR optimization and validation

To check the sensitivity of TL-qPCR, we tested serial diluted synthetic oligos samples. Briefly, 2 µL 10× serial diluted synthetic oligos samples with concentrations ranging from 1 nM to 0.1 pM were mixed with 2 µL 10× ligation linker oligos solution. TL-qPCR was then performed as described above. To test the effect of temperature and RNA modifications on SplintR ligation efficiency, template ligation reactions were performed at different temperatures (32°C, 37°C, 42°C, or 47°C) using ligation linker oligos targeting the m^1^A modification site or sites away from the m^1^A site. TL-qPCR was then performed as described above.

## SUPPLEMENTAL MATERIAL

Supplemental material is available for this article.

## Figures and Tables

**Wen Zhang:**
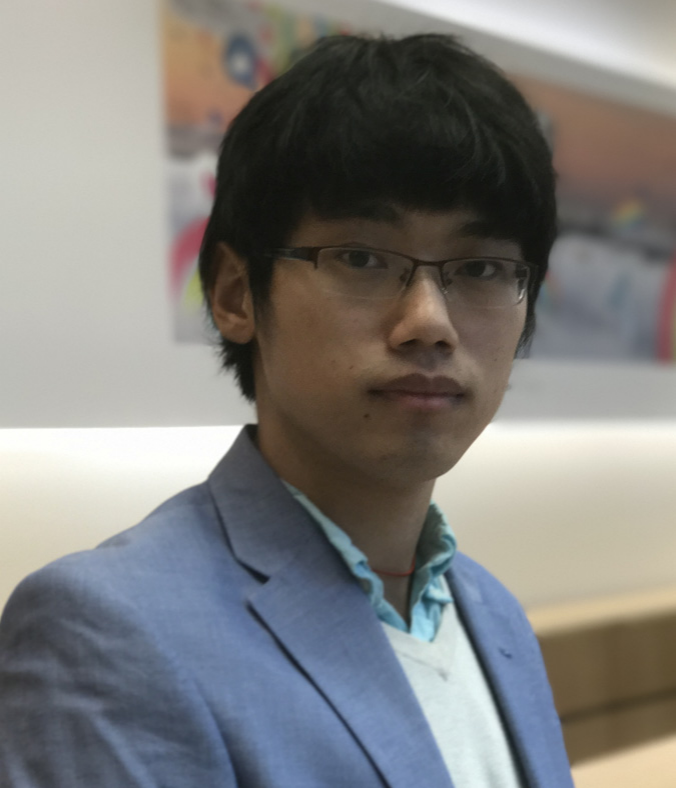


## MEET THE FIRST AUTHOR

***Meet the First Author(s)* is an editorial feature within *RNA*, in which the first author(s) of research-based papers in each issue have the opportunity to introduce themselves and their work to readers of *RNA* and the RNA research community. Wen Zhang is the co-first author of this paper, “Quantification of tRNA m^1^A modification by templated-ligation qPCR,” along with Hankui Chen. Wen is a postdoc student in Dr. Tao Pan's laboratory at the University of Chicago. His research focus is RNA modification and microbiome metabolites.**

**What are the major results described in your paper and how do they impact this branch of the field?**

We developed a template ligation and qPCR-based quantification method for tRNA m^1^A modification and tRNA abundance. Our method requires a small amount of input RNA and can be done in a matter of hours. It will be useful for the quantification of tRNA abundance and m^1^A modification when input samples and time are limited, such as when testing clinical samples.

**What led you to study RNA or this aspect of RNA science?**

Previous studies of our lab showed that m^1^A modification levels in specific tRNAs can be used as biomarkers for clinical prognosis such as the development of COVID-19 severity. Since clinical samples are often limited by the amount, and the tested results should be available as soon as possible, we wanted to develop a sensitive and fast method for the quantification of m^1^A modification.

**During the course of these experiments, were there any surprising results or particular difficulties that altered your thinking and subsequent focus?**

The Dimroth rearrangement of m^1^A modification in control samples caused some problems at the beginning. However, when we applied a new synthesis method to avoid Dimroth rearrangement, we saw nearly no ligated product when we used 100% m^1^A-modified oligo as input. This means that the problem of base-pairing at even one nucleotide caused by m^1^A modification is efficient in blocking SplintR template ligation.

**What are some of the landmark moments that provoked your interest in science or your development as a scientist?**

It was during high school orientation, when the principal of a local university gave a talk about “quantum chemistry.” Although it was hard to understand the talk as a first-year high school student, the talk did make me think more about the materials that make up the world.

**What are your subsequent near- or long-term career plans?**

My short-term goal is to finish the project I have been working on for a few years and probably find a second postdoc position if it is necessary. My long-term career plan is to find an academic job.

**How did you decide to work together as co-first authors?**

Hankui was the research scientist in the lab and was very good at qPCR. I was the postdoc in the lab and had studied tRNA modifications for a long time. We both agreed that combining our strengths would make this project move forward smoothly.
